# Depression and Anxiety Disorders among Hospitalized Women with Breast Cancer

**DOI:** 10.1371/journal.pone.0129169

**Published:** 2015-06-02

**Authors:** Neomi Vin-Raviv, Tomi F. Akinyemiju, Sandro Galea, Dana H. Bovbjerg

**Affiliations:** 1 Rocky Mountain Cancer Rehabilitation Institute, School of Sport and Exercise Science, University of Northern Colorado, Greeley, Colorado, United States of America; 2 Department of Epidemiology, University of Alabama School of Public Health, Birmingham, Alabama, United States of America; 3 Boston University School of Public Health, Boston, Massachusetts, United States of America; 4 Biobehavioral Oncology Program, Department of Psychiatry, University of Pittsburgh Cancer Institute, Pittsburgh, Pennsylvania, United States of America; 5 Biobehavioral Oncology Program, Department of Psychology, University of Pittsburgh Cancer Institute, Pittsburgh, Pennsylvania, United States of America; 6 Biobehavioral Oncology Program, Department of Behavioral & Community Health Sciences, University of Pittsburgh Cancer Institute, Pittsburgh, Pennsylvania, United States of America; Institute of Psychiatry, UNITED KINGDOM

## Abstract

**Purpose:**

To document the prevalence of depression and anxiety disorders, and their associations with mortality among hospitalized breast cancer patients.

**Methods:**

We examined the associations between breast cancer diagnosis and the diagnoses of anxiety or depression among 4,164 hospitalized breast cancer cases matched with 4,164 non-breast cancer controls using 2006-2009 inpatient data obtained from the Nationwide Inpatient Sample database. Conditional logistic regression models were used to compute odds ratios (ORs) and 95% confidence intervals (CI) for the associations between breast cancer diagnosis and diagnoses of anxiety or depression. We also used binary logistic regression models to examine the association between diagnoses of depression or anxiety, and in-hospital mortality among breast cancer patients.

**Results:**

We observed that breast cancer cases were less likely to have a diagnosis of depression (OR=0.63, 95% CI: 0.52-0.77), and less likely to have a diagnosis of anxiety (OR=0.68, 95% CI: 0.52-0.90) compared with controls. This association remained after controlling for race/ethnicity, residential income, insurance and residential region. Breast cancer patients with a depression diagnosis also had lower mortality (OR=0.69, 95% CI: 0.52-0.89) compared with those without a depression diagnosis, but there was no significant difference in mortality among those with and without anxiety diagnoses.

**Conclusion:**

Diagnoses of depression and anxiety in breast cancer patients were less prevalent than expected based on our analysis of hospitalized breast cancer patients and matched non-breast cancer controls identified in the NIS dataset using ICD-9 diagnostic codes. Results suggest that under-diagnosis of mental health problems may be common among hospitalized women with a primary diagnosis of breast cancer. Future work may fruitfully explore reasons for, and consequences of, inappropriate identification of the mental health needs of breast cancer patients.

## Introduction

Recent progress in cancer treatment has markedly improved the prognosis for many breast cancer patients. Although breast cancer remains the most common cancer among women in the United States, accounting for over 230,000 new cases and 39,000 deaths in 2013, death rates are now about one third of their levels in 1990 [1]. The improvements in the quality and variety of cancer treatment modalities, coupled with an increase in America’s aging population [2], have also led to significantly higher numbers of breast cancer survivors in the past decade. This progress brings new challenges. More women with breast cancer are living longer with uncertainty about the future, the burden of treatment [3], and the wide range of emotional, social and psychological difficulties their situation brings [4]. Previous studies have found that 35%-38% of all breast cancer patients experience significant emotional distress, anxiety, and/or depression following their diagnosis [5–9]. Such co-occurring mental health problems can influence cancer progression [10,11], and decrease survival among patients with cancer [12,13].

There are multiple barriers to identifying and diagnosing mental health disorders among cancer patients. These include patient-level barriers such as fear of the stigma of a psychiatric diagnosis, and concerns that a focus on a psychiatric disorder may distract from oncological treatment [14]. At the provider level, barriers may include lack of time to make a mental health assessment, reluctance to shift focus of treatment from cancer, and limited availability of mental health specialists collaborating with oncologists on treatment plans [15,16]. At the institutional level, low priority, fragmented or poorly coordinated psychosocial services, and ineffective communication across clinical settings may contribute to low levels of mental health diagnoses among cancer patients [15]. Despite those considerations, to the best of our knowledge very few studies have examined the prevalence of mental health diagnoses among women with breast cancer who have been admitted to the hospital [17–19], an indication of more severe disease. Hospitalized breast cancer patients are likely at greater risk for psychological distress compared with their outpatient counterparts as a result of being admitted, while also potentially experiencing the personal and provider level barriers that may limit diagnosis of mental illness [20].

When considering mental health disorders among cancer patients, the research literature has typically emphasized the potential for depression and anxiety. However despite many years of research, the prevalence of depression and anxiety disorders among cancer patients is still subject to much debate[21,22], partly due to the use of self-reported scales rather than structured diagnostic interviews in most prior studies [21,23]. Structured diagnostic interviews, are considered the “gold standard” in the assessment of mental health disorders, but are less often used due to time constraints and demands of the clinical routine. Two recent meta-analyses that focused on psychiatric interviews found that depression and anxiety disorders defined by the Diagnostic and Statistical Manual of Mental Disorders (DSM) or International Classification of Diseases (ICD) criteria are less common in patients with cancer than previously thought[24,25]. Mitchell and colleges[24] demonstrated that when defined by the DSM or ICD criteria, the meta-analytical pooled prevalence was 16.5% for depression and 9.8% for anxiety disorders, without a significant difference between palliative-care and non-palliative-care settings. Walker et al.[25] also observed that current depression prevalence ranged from 5% to 16% in outpatients, 4% to 14% in inpatients, 4% to 11% in mixed outpatient and inpatient samples and 7% to 49% in palliative care. These estimates are much lower than previous studies using self-reported scales, which have found that 35%-38% of all breast cancer patients experience significant emotional distress, anxiety, and/or depression following their diagnosis[5–9]. The question of whether or not existing clinical diagnostic criteria are ideal in cancer settings is still unclear[23,24], and most studies have used the diagnostic criteria from DSM to define case-ness for mental disorders[24,25] with little attention given to the ICD criteria[26].

The aim of the present study was to assess the prevalence of clinically diagnosed depression and/or anxiety among hospitalized patients with a primary breast cancer diagnosis using ICD-9 clinical diagnostic codes, and to examine associations between a diagnosis of depression or anxiety with mortality.

## Methods

### Study Design and Data Source

A cross-sectional study of hospital inpatient admissions was conducted. We obtained data for this study from the Healthcare Cost and Utilization Project (HCUP) produced Nationwide Inpatient Sample (NIS) database [27]. The NIS is a comprehensive database of hospital inpatient stays that represents a 20% stratified sample of community hospitals in the United States, including public hospitals and academic medical centers. The NIS includes data from over 1,000 hospitals in the US from 44 states currently participating in HCUP; in 2009 these hospitals covered about 96% of the entire US population[27]. The NIS contains data from over 7 million hospital stays per year, with weighted estimates of over 36 million hospitalizations nationally per year. The large size of the dataset makes it ideal for studies of associations among diseases such as breast cancer and mental health disorders. NIS contains clinical and non-clinical data elements for each hospital stay including primary and secondary diagnoses and procedures, demographics, severity measures and length of stay. Further details about the NIS can be obtained from http://www.hcup-us.ahrq.gov/nisoverview.jsp.

### Cohort assembly

We restricted our sample to women between ages 40 and 85 years, who were admitted to one of the HCUP participating hospitals. For the case-control analysis, we randomly selected a 10% sample of NIS data from 2006–2009 without replacement (n = 943,888), and created matched dataset with breast cancer cases and matched controls. For the mortality analysis, we identified all breast cancer cases diagnosed in the NIS between 2006 and 2009, and identified all in-hospital deaths among those cases. We used the International Classification of Disease, ninth edition (ICD-9 codes: 174.0–174.9, 175.0, 175.9, 198.2, 172.5, 173.5, 232.5, 216.5, 233.0) to identify hospital admissions for women with a primary diagnosis of breast cancer.

### Matching

For the analysis of the association between mental health diagnosis and breast cancer diagnosis, we selected women without a primary breast cancer diagnosis and 1:1 matched them to women with primary breast cancer diagnosis on age at admission, length of stay, number of comorbidities, and discharge disposition using the % gmatch macro- a greedy matching algorithm published by the Mayo Clinic (Mayo Clinic, MN) [28]. This algorithm matches cases to controls by randomly sorting the cases and controls, matching the first case to the closest control, and moving on to the second case and matching to the closest control among the remaining controls until all cases have been matched. This approach is called ‘greedy’ because the consideration of the best match for a case is made at each match, without consideration of its future impact on the total matching.

### Patient and hospital characteristics

We examined the NIS data from 2006–2009 for both cases and controls in order to evaluate mental health diagnoses. To achieve power, we created *a priori* groupings of depression and anxiety diagnoses based on ICD-9-defined categories and ad hoc clinical considerations. For this analysis, a clinical diagnosis of depression included ICD-9 codes for major depression (codes 296.2, 296.3), depressive disorder NOS (code 311); a clinical diagnosis of anxiety included ICD-9 codes for anxiety disorder NOS (code 300.0), acute reaction to stress (code 308) and posttraumatic stress disorder (PTSD) (codes 309.81, 309.82, 309.83, 309.89).

In addition to age, race/ethnicity, residential income, insurance type, residential region, length of stay, stage at admission (for breast cancer cases), we also created a modified Deyo comorbidity score based on ICD-9 codes for major comorbid conditions. These included myocardial infarctions, congestive heart failure, peripheral vascular disease, cerebrovascular disease, dementia, chronic pulmonary disease, rheumatic disease, peptic ulcer disease, mild liver disease, diabetes mellitus with or without chronic complications, hemiplegia or paraplegia, renal disease, moderate or severe liver disease, and HIV/AIDS. We summed the number of these conditions for each patient into a single comorbidity score to reflect the overall comorbidity of the patient (COM). The Charleston index as modified by Deyo was previously examined in the NIS database [29]. Length of stay (LOS) was calculated by subtracting the admission date from the discharge date [29], with same-day stays coded as 0.

Residential income was estimated based on the median household income at the zip-code level, and was classified into quartiles with values ranging from 1 for the poorest zip code to 4 for the wealthiest zip code. Residential region was categorized at the county level into large metropolitan areas (metropolitan areas with 1 million residents or more), small metropolitan areas (metropolitan areas with less than 1 million residents), micropolitan areas (Non-metropolitan areas adjacent to metropolitan areas) and non-metropolitan or micropolitan areas (noncore areas with or without own town). These designations were developed by HCUP based on the 2003 version of the Urban Influence Codes (UIC) [30].

The NIS does not include directly coded data on stage of presentation, so we created a new variable using the clinical criteria of disease staging based on ICD-9 codes. Metastatic disease was assigned when the ICD-9 code indicated metastatic disease to other organs (196.0–196.2, 196.5–196.9, 197.0–197.8, 198.80–198.89 and 199.00–199.18), non-metastatic disease was assigned when those specific codes were absent, and in-situ was assigned when ICD-9 code 2330 was present. Disease staging has been evaluated and validated in previous studies [31], and was previously examined in the NIS database [29].

### Outcomes

We were interested in examining two separate outcomes: 1) Diagnoses of mental health disorders in breast cancer inpatients comparing cases with matched non-breast cancer controls; and 2) In-hospital mortality among breast cancer patients, defined as death during hospitalization using a sample of breast cancer cases. The NIS only contains inpatient data; therefore deaths occurring after discharge are not captured. The Columbia University Medical Center Institutional Review Board exempted this study from ethical review.

### Statistical Analysis

In the matched analysis of breast cancer patients and non-breast cancer controls, we used paired t-tests and McNemar’s test for descriptive analyses, and Bowker’s test of symmetry for paired variables with multiple levels. We examined the distribution of non-clinical characteristics between groups and computed p-values, taking into account the matched design. We used conditional logistic regression analyses to generate odds ratios and p-values for the association between depression and anxiety and odds of breast cancer adjusting for other variables. This allowed us to account for the matching design. We presented two models for this analysis; the first model adjusted for race/ethnicity, and the second model adjusted for race/ethnicity, residential income, insurance type and residential region. In the analysis of in-hospital mortality among breast cancer patients, we used simple descriptive statistics to compute the number of deaths in each category. We used binary logistic regression to examine the association between depression and anxiety, and odds of in-hospital mortality (yes/no) among breast cancer patients, adjusting for a priori specified confounders. We present three models for this analysis; the first model adjusts for race/ethnicity, age at admission and stage at admission, the second model additionally adjusts for residential income, region and insurance, while the third model additionally adjusts for length of stay and number of comorbidities. Longer hospital length of stay and higher number of comorbidities are associated with being sicker, and higher mortality. We assessed for confounding by entering potential confounders individually into models including the main predictive variables. We used SAS software, version 9.3 (SAS Institute, Cary, NC) for all statistical analyses, and included sampling weights to account for the stratified study design.

## Results

Our sample identified 6,512 women aged 40–85 with a primary diagnosis of breast cancer between 2006 and 2009. Of these, the algorithm was able to match 4,164 with non-breast cancer cases as controls. Cases and controls were similar on all matched (age at admission, length of stay, number of comorbidities, and discharge disposition) characteristics (Table 1), and the racial/ethnic distribution was similar for both groups. However, there were significant differences between the groups in terms of residential income, insurance type, and residential region. About 29% of the cases resided in a high residential income area, compared with 20% of controls (p<0.001), 49% of the cases had private insurance compared with 41% of the controls (p<0.001), and 54% of cases resided in a large metropolitan area compared with 47% of controls (p<0.001). About 1.8% of the cases died during hospitalization, compared with 1.6% of the controls (p = 0.06).

**Table 1:** Demographic and Patient Characteristics comparing Breast Cancer Cases and Matched Controls, Nationwide Inpatient Sample, 2006–2009* ^+^.

	Cases	Matched Controls	p-value
	(N = 4,164)	(N = 4,164)	
	N (%)/Mean (SD)	N (%)/ Mean (SD)	
Age at admission—years (matched)	60.9 (12.16)	60.9 (12.17)	0.98
Length of Stay—days (matched)	2.47 (2.84)	2.46 (2.79)	0.23
Number of Co-morbidities (matched)	0.23 (0.51)	0.23 (0.52)	0.26
Discharge Disposition (matched)			0.46
Routine Discharge	3005 (72.2)	3006 (72.2)	
Discharged to Skilled Nursing Facility	1025 (24.6)	1035 (24.9)	
Expired	76 (1.8)	69 (1.7)	
Discontinued Care	3 (0.1)	3 (0.1)	
Other	55 (1.3)	51 (1.2)	
Race/Ethnicity			0.55
White	2,375 (73.4)	2,387 (75.5)	
Black	407 (12.9)	381 (12.1)	
Hispanic	203 (6.4)	229 (7.3)	
Other	167 (5.3)	163 (5.2)	
Residential income			<0.001
First Quartile-Lowest	958 (23.5)	1,133 (27.9)	
Second Quartile	977 (23.9)	1,138 (28.0)	
Third Quartile	963 (23.7)	977 (24.1)	
Fourth Quartile-Highest	1,174 (28.8)	812 (20.0)	
Insurance Type			<0.001
Medicare	1,554 (37.3)	1,790 (42.9)	
Medicaid	333 (8.0)	336 (8.1)	
Private	2,055 (49.4)	1,711 (41.1)	
Other	222 (5.3)	327 (7.9)	
Residential Region			<0.001
Large Metro (>1mil residents)	2,237 (53.7)	1,974 (47.4)	
Small Metro (<1 mil residents)	1,059 (25.4)	1,165 (27.9)	
Micropolitan (Adjacent to metro)	423 (10.2)	538 (12.9)	
Non-metro or micropolitan	277 (6.7)	354 (8.5)	
Died during Hospitalization			0.06
No	4,091 (98.2)	4096 (98.4)	
Yes	73 (1.75)	68 (1.63)	
Stage at presentation			
In-Situ	530 (12.7)	-	
Non-Metastatic	2,363 (56.7)	-	
Metastatic	1,271 (30.5)	-	

*Percentages may not add up to 100% due to rounding.

^+^Cell values may not add up to total due to missing data. There were 1012 cases and 1014 controls with missing data on race/ethnicity, and 92 cases and 104 controls with missing data on residential income.

Among breast cancer cases, 355 (8.5%) women had a clinical diagnosis of depression, while 172 (4.1%) had a clinical diagnosis of anxiety (Table 2). Among controls, 579 (13.9%) women had a depression diagnosis, and 246 (5.9%) had an anxiety diagnosis. Only 83 women had a clinical diagnosis of both depression and anxiety. In unadjusted analysis (Table 2), there was no association between race/ethnicity and breast cancer diagnosis, however increasing residential income was associated with increased odds of breast cancer diagnosis (highest vs. lowest residential income OR = 1.7, 95% CI = 1.52–1.96). Compared with private insurance, Medicare (OR = 0.51, 95% CI: 0.44–0.59), Medicaid (OR = 0.82, 95% CI: 0.69–0.97) and other (OR = 0.57,95% CI: 0.47–0.68) insurance types were associated with lower odds of breast cancer diagnosis, and residence outside of a large metropolitan area was also associated with lower odds of breast cancer diagnosis (Non-metro/micropolitan residence OR = 0.68, 95% CI: 0.58–0.82).

**Table 2:** Association between breast cancer diagnosis and clinically diagnosed anxiety and depression among matched cases and controls, Nationwide inpatient sample, 2006–2009^+^.

	Cases	Controls	Unadjusted	Adjusted	Fully adjusted
	N = 4,164	N = 4,164	OR (95% CI)	OR (95% CI) ^1^	OR (95% CI) ^2^
**Diagnosed Depression**					
No	3,809	3,585	Ref	Ref	Ref
Yes	355	579	0.58 (0.50–0.66)**	0.58 (0.48–0.70)***	0.63 (0.52–0.77)***
**Diagnosed Anxiety**					
No	3,992	3,918	Ref	Ref	Ref
Yes	172	246	0.67 (0.55–0.83)**	0.65 (0.50–0.84)**	0.68 (0.52–0.90)**
**Race/Ethnicity**					
White	2,375	2,387	Ref	Ref	Ref
Black	407	381	0.95 (0.81–1.14)	1.01 (0.84–1.20)	1.08 (0.88–1.31)
Hispanic	203	229	1.23 (0.97–1.55)	0.80 (0.63–1.01)	0.79 (0.61–1.02)
Other	167	163	1.01 (0.79–1.30)	0.97 (0.76–1.26)	0.82 (0.63–1.08)
**Residential Income**					
Q1-Lowest	958	1,133	Ref		Ref
Q2	977	1,138	1.01 (0.89–1.14)		0.97 (0.82–1.16)
Q3	963	977	1.15 (1.07–1.30)		0.97 (0.81–1.18)
Q4-Highest	1,174	812	1.73 (1.52–1.96)		1.36 (1.12–1.64)***
**Insurance Type**					
Private	2,055	1,711	Ref		Ref
Medicare	1,554	1,790	0.51 (0.44–0.59)**		0.58 (0.47–0.70)**
Medicaid	333	336	0.82 (0.69–0.97)*		0.95 (0.75–1.21)*
Other	222	327	0.57 (0.47–0.68)*		0.62 (0.48–0.80)*
**Residential Region**					
Large metro	2,237	1,974	Ref		Ref
Small metro	1,059	1,165	0.79 (0.72–0.88)*		0.87 (0.75–1.01)
Micropolitan	423	538	0.68 (0.59–0.79)*		0.72 (0.58–0.91)*
Not metro or micro	277	354	0.68 (0.58–0.82)*		0.72 (0.58–0.91)*

^1^Adjusted for race/ethnicity

^2^Adjusted for race/ethnicity, residential income, insurance and residential region

*** p-value <0.001;

** p-value <0.01,

* p-value <0.05

^+^Cell values may not add up to total due to missing data. There were 1012 cases and 1014 controls with missing data on race/ethnicity, and 92 cases and 104 controls with missing data on residential income.

Adjusting for race/ethnicity did not attenuate the associations between depression and anxiety diagnoses and breast cancer. Furthermore, in the fully adjusted model, the associations between depression diagnoses (OR = 0.63, 95% CI: 0.52–0.77) or anxiety diagnoses (OR = 0.68, 95% CI: 0.52–0.90) and breast cancer remained; breast cancer cases were 37% less likely to have depression diagnoses, and 32% less likely to have anxiety diagnoses compared with matched non-breast cancer controls after additionally adjusting for residential income, insurance type and residential region.

Among the 64,425 breast cancer cases diagnosed in the NIS between 2006 and 2009, there were 1,146 in-hospital deaths (Table 3). Black (OR = 1.65, 95% CI: 1.39–1.96) and Hispanic (OR = 1.33, 95% CI: 1.06–1.68) cases were more likely to experience in-hospital deaths compared with white cases in unadjusted analysis. Patients with depression diagnoses had lower mortality compared with those without a depression diagnosis (OR = 0.69, 95% CI: 0.52–0.89), but there was no significant difference in mortality among those with anxiety diagnoses (OR = 1.05, 95% CI: 0.78–1.40). Metastatic breast cancer patients were more than 11 times more likely to experience in-hospital deaths compared with non-metastatic patients (OR = 11.13, 95% CI: 9.44–13.12). Residents of higher residential income areas were much less likely to die in the hospital compared with residents of low residential income areas (highest residential income OR = 0.52, 95% CI: 0.44–0.62), and cases with Medicaid (OR = 1.61, 95% CI: 1.33–1.96) or other (OR = 3.35, 95% CI: 2.79–4.03) insurance types had much higher in-hospital deaths compared with cases with private insurance. In addition, residents of areas outside of large metropolitan areas had higher in-hospital deaths compared with residents of large metro areas (Non-metro/micropolitan residence OR = 1.60, 95% CI: 1.26–2.04).

**Table 3:** Association between in-hospital mortality and clinically diagnosed anxiety and depression among breast cancer cases, Nationwide inpatient sample, 2006–2009^+^.

Variable, n (%)	Deaths	Cases	Unadjusted	Adjusted	Additionally Adjusted	Fully Adjusted
	(N = 1,146)	(64,425)	OR (95% CI)	OR (95% CI) ^1^	OR (95% CI) ^2^	OR (95% CI) ^3^
**Diagnosed Depression**						
No	1,084 (95)	59,375 (92)	Ref	Ref	Ref	Ref
Yes	62 (5)	4,937 (8)	0.69 (0.52–0.89)	0.74 (0.55–0.98)**	0.76 (0.57–1.01)	0.68 (0.51–0.91)*
**Diagnosed Anxiety**						
No	1,098 (96)	61,731 (96)	Ref	Ref	Ref	Ref
Yes	48 (4)	2,581 (4)	1.05 (0.78–1.40)	1.08 (0.78–1.50)	1.09 (0.78–1.52)	0.97 (0.69–1.37)
**Race/Ethnicity**						
White	617 (54)	37,186 (58)	Ref	Ref	Ref	Ref
Black	168 (15)	6,208 (10)	1.65 (1.39–1.96)	1.42 (1.19–1.69)*	1.32 (1.09–1.60)*	1.07 (0.88–1.32)
Hispanic	86 (8)	3,902 (6)	1.33 (1.06–1.68)	1.29 (1.02–1.62)*	1.14 (0.89–1.45)	1.15 (0.89–1.48)
Other	50 (4)	3,386 (5)	0.89 (0.66–1.19)	0.92 (0.68–1.23)	0.92 (0.67–1.24)	0.87 (0.64–1.21)
**Stage at Diagnosis**						
In-Situ	3 (0.3)	8,173 (13)	0.08 (0.02–0.24)	0.10 (0.03–0.32)***	0.11 (0.03–0.34)***	0.07 (0.02–0.31)***
Non-Metastatic	168 (15)	36,319 (56)	Ref	Ref	Ref	Ref
Metastatic	975 (85)	19,820 (31)	11.13 (9.44–13.12)	11.11 (9.23–13.37)***	11.16 (9.23–13.49)***	9.65 (7.96–11.70)***
**Residential Income**						
Q1-Lowest	322 (28)	14,304 (23)	Ref		Ref	Ref
Q2	313 (27)	14,880 (24)	0.93 (0.70–1.09)		1.01 (0.85–1.23)	1.01 (0.84–1.24)
Q3	264 (23)	15,131 (24)	0.77 (0.65–0.91)		1.02 (0.84–1.25)	1.05 (0.85–1.28)
Q4-Highest	220 (19)	18,539 (30)	0.52 (0.44–0.62)		0.78 (0.63–0.97)**	0.79 (0.63–0.98)**
**Insurance Type**						
Private	481 (42)	31,288 (49)	Ref		Ref	Ref
Medicare	374 (33)	24,424 (38)	0.99 (0.86–1.14)		0.58 (0.47–0.72)***	0.54 (0.44–0.66)***
Medicaid	134 (12)	5,444 (8)	1.61 (1.33–1.96)		1.02 (0.81–1.29)	0.77 (0.60–0.98)*
Other	157 (14)	3,156 (5)	3.35 (2.79–4.03)		2.06 (1.65–2.59)***	1.86 (1.47–2.35)***
**Residential Region**						
Large metro	428 (37)	27,606 (43)	Ref		Ref	Ref
Small metro	208 (18)	11,953 (19)	1.13 (0.95–1.33)		1.06 (0.87–1.29)	1.12 (0.91–1.37)
Micropolitan	104 (9)	4,754 (7)	1.42 (1.14–1.76)		1.32 (1.01–1.74)	1.44 (1.09–1.89)**
Not metro or micro	79 (7)	3,209 (5)	1.60 (1.26–2.04)		1.62 (1.20–2.19)*	1.79 (1.32–2.43)**

^1^Adjusted for age at admission, race/ethnicity and stage

^2^Adjusted for age at admission, race/ethnicity, stage, residential income, region, insurance

^3^Adjusted for age at admission, race/ethnicity, stage, residential income, region, insurance, LOS, COM

*** p-value <0.001;

** p-value <0.01,

* p-value <0.05

^+^Cell values may not add up to total due to missing data. There were a total of 13,743 cases with missing information on race/ethnicity; 113 cases with missing information on depression, anxiety and stage at diagnosis, insurance, and region; and 1571 cases with missing data on residential income

After adjusting for race/ethnicity and stage at diagnosis, the inverse association between depression diagnoses and breast cancer mortality remained significant (OR = 0.74, 95% CI: 0.55–0.98), and anxiety diagnoses remained non-significant. After additionally adjusting for residential income, residential region and insurance, the association between depression diagnoses and mortality became non-significant (OR = 0.76, 95% CI: 0.57–1.01). However, in the fully adjusted model, which includes length of stay and number of comorbidities, the association between depression and mortality became significant (OR = 0.68, 95% CI: 0.51–0.91), while anxiety remained non-significant (OR = 0.97, 95% CI: 0.69–1.37). In addition, in the fully adjusted model, the racial/ethnic differences in mortality became non-significant for blacks (OR = 1.07, 95% CI: 0.88–1.32), and Hispanics (OR = 1.15, 95% CI: 0.89–1.48).

## Discussion

Our analysis of the 2006–2009 NIS data of hospitalized breast cancer patients and matched non-breast cancer controls indicated that clinical diagnoses of depression and anxiety were less prevalent among breast cancer patients compared with non-breast cancer controls. These differences persisted after adjusting for potential confounders such as age at admission, length of stay, number of comorbidities, and discharge disposition through the matched design, and on race/ethnicity, residential income, health insurance and region of residence in the analysis. Additionally, we observed that a diagnosis of depression was associated with significantly lower mortality among breast cancer patients compared with non-breast cancer matched controls, but we found no difference in mortality with a diagnosis of anxiety.

Although few population-based studies have examined the prevalence of clinically diagnosed mental health disorders among cancer patients, those have mostly been in mixed inpatient and outpatients records [17,32] or only outpatients [18]. To put the findings of our study into context, the prevalence found here is much higher than observed in one study of colorectal cancer outpatients [18]. Zhang and Cooper reported that the clinical diagnostic rates among colorectal cancer outpatients who were Medicare beneficiaries in 2002 was 1.5% for depressive disorders, and 1.1% for anxiety disorders, and in non-cancer outpatients approached 2.5% for depressive disorders and 1.6% for anxiety disorders [18]. In contrast, another study documented higher prevalence rates of mental health disorders (20%) among male Medicare beneficiaries with localized prostate cancer [32], while Erale and colleagues [17] reported a mental health disorder prevalence of 33.5% among cancer survivors compared to 30% in the non-cancer control group. These differences may be due to methodological limitations such as differences in the study population, time period, or use of a more broad-based definition of mental health diagnoses.

The lower rates of diagnosed depression and anxiety observed in our study among hospitalized breast cancer patients compared with matched non-cancer controls was unexpected and raises questions about research focused on mental health in cancer patients. Despite many years of research, prevalence of depression and anxiety in patients with cancer is still subject to much debate [21,22]. Previous studies have reported prevalence estimates ranging from 1.5% to 50% for depression [22,33], and the prevalence for anxiety disorders has been estimated to be 6% to 23% [34]. The wide range of estimates may stem from wide variability in the case mix present both within and across study samples (e.g., type of cancer diagnosis, disease stage at diagnosis, type of treatment received, age, race and ethnicity, gender, and time point in the survivorship trajectory when mental health issues are assessed) [22,33]. In addition, most of the previous studies were based on self-reported questionnaire data rather than diagnostic instruments [21,24,25,33], and were lacking a consistent definition of depression (e.g., major depression from the Diagnostic and Statistical Manual of Mental Disorder, DSM or depressive episode from the International Classification of Diseases, ICD) [22,33]. Studies that used expert interviewers (psychiatrist or clinical psychologist) or the Structured Clinical Interview for Diagnosis (SCID) to diagnose depression were more consistent in their findings and reported lower prevalence estimates of depression than other studies [35, 36]. Similar patterns have been reported in the literature on cancer-related PTSD. When the SCID was administrated the prevalence of cancer-related PTSD ranged between 0%- 9% [37]. In contrast, in studies that utilized self-reported questionnaires, the prevalence rates for PTSD were as high as 55% [23]. Utilization of the ICD-9 to diagnose mental health disorders may also have contributed to lower observed prevalence. This is because the ICD-9 diagnostic criteria are more stringent and factors relating to the coding and reporting of ICD-9 diagnoses may also contribute to the low rate of mental health diagnoses [18,38]. Nevertheless, our results are consistent with previous studies that utilized the ICD-9 criteria among different cancer populations [18]. Additional studies are needed to validate existing prevalence estimates using different diagnostic criteria for mental health problems in cancer patients.

Alternative explanations for the low prevalence of depression and anxiety observed in our study among hospitalized breast cancer patients need to be considered, including one or more diagnostic barriers at the individual, provider or institutional levels. A recent study by Walker et al [39] demonstrated that while the prevalence of major depression among cancer patients in Scotland ranged from 5.6% to 13.1%, the majority of those cancer patients with depression were undertreated (73%). Additionally, Zhang et al [40] observed that hospitalized breast cancer patients were more likely to have elective admissions (66%) compared with the general inpatient population (25%) who present with other chronic diseases. Consequently, primary breast cancer patients have shorter lengths of stay, fewer diagnoses, but more procedures on average, and significantly lower total hospital charges [40]. This raises the possibility that breast cancer patients are being admitted for specific, targeted purposes that may preclude routine mental health screening. In addition, biological responses to cancer or its treatment can result in symptoms associated with depression [25,41] or anxiety [21]. Finally, as highlighted in a recent pilot study of hospitalized cancer patients, since access to patients was not possible until after admissions processes had concluded, it was not uncommon for the commencement of diagnostic and treatment procedures to eliminate opportunities for completing the mental health/distress screenings [20]. Other studies suggest that oncologists may have difficulty detecting psychiatric morbidities [15,16,42]. Earle and colleagues [17] examined mental health care utilization claims using the ICD-9 psychiatric diagnosis criteria in a cohort of cancer survivors and found that while the majority of anxiety related diagnosis were assigned by a primary care physician (45%) and mental health professional (27%), only 1% were assigned by oncologists. This could be due to patient level factors such as fear that a mental health diagnosis would distract from oncological treatment leading to underreporting of depression or anxiety symptoms; it could also be due to provider and/or institutional level factors such as a strict focus on the cancer diagnosis to the exclusion of other comorbidities, and lack of skilled mental health personnel well integrated into clinical care routine for complex diseases such as breast cancer.

Our results also indicated that hospitalized breast cancer patients with a clinical diagnosis of depression had lower mortality rates compared with matched patients without a depression diagnosis. This finding stands in apparent contrast to previously reported associations between depression and higher mortality among cancer patients [12,13]. In cancer patients, depression and anxiety have been associated with non-adherence to medication [33,43], increased use of emergency and medical inpatient services [44]. Our current study findings are consistent with those by Zhang et al. using the NIS data to model the impact of concurrent mental health comorbidities on outcomes such as hospital length of stay, mortality and hospital charges [18]. The authors observed that among primary breast cancer patients with secondary diagnoses of a mental health disorder, hospital length of stay was reduced by almost 20%, but total hospital charges increased by about 3%. In addition, in-hospital mortality was 30% lower compared with the general breast cancer inpatient population. One major difference between the two studies was that Zhang et al. used one broad category of mental disorder, while our study separated depression and anxiety disorders [18]. However, our findings that in-hospital mortality was lower among patients diagnosed with a mental disorder compared with those undiagnosed are quite similar.

Evidence about the association between depression and survival in cancer populations, has been inconsistent [12,45–49]. Recent meta-analytic analyses of data from prospective studies of patients with various types of cancer concluded that depression at the time of diagnosis is associated with increased mortality [12,13]. However, the majority of studies included mixed patient samples with various tumors, and differences in the timing and nature of depression assessment. The majority of existing studies using self-reported questionnaires failed to identify an association between depression and survival [50–54]. In contrast, two studies based on clinician diagnoses using ICD-8 [55] and ICD-9 [19] codes demonstrated significant relationships between depression and survival in breast cancer patients, such that a diagnosis of depression was associated with a 1.3–1.4-fold decrease in survival [19,55]. Our observation that hospitalized breast cancer patients were less likely than hospitalized controls to be diagnosed with depression and anxiety, and that breast cancer patients who were diagnosed were less likely to experience in-hospital mortality suggests the need for a better and more integrated cancer care approach. However, not all hospitals have the necessary resources to provide oncology and mental health services. This may have contributed to our observation that breast cancer mortality was higher among residents outside of large metropolitan areas, further highlighting the importance of developing an integrated strategy at the institutional level to ensure that both the physical and mental health needs of cancer patients are considered.

This study was based on data from a large administrative database that has several strengths and limitations. The NIS is a large representative sample of hospitalizations across the US, providing a unique opportunity to examine two relatively rare diseases simultaneously. The matched case-control design also helps to minimize measured and potential unmeasured confounders that may distort the association between breast cancer diagnosis and mental health diagnoses. However, the dataset is limited to in-patient hospitalizations and in-hospital deaths and therefore does not capture patients that were discharged home or to a hospice before deaths. In addition, since the dataset includes only de-identified patient records, it is not possible to exclude duplicate records if the same patient was admitted multiple times in the same year. Zhang et al. tried to identify possible duplicate situations by using zip code, income, and location data, however they were unable to exclude duplicate records [18]. Another limitation is that previous studies have demonstrated that the presence of depression before the cancer diagnosis may increases the risk of cancer- and all-cause mortality[19], similar to the effects of depression on risk of morbidity and mortality in several other chronic diseases, including AIDS [56] and cardiovascular disease [57]. In addition, the NIS data does not include any information on pre-existing conditions or cancer-specific clinical variables such as hormone receptor status. We were therefore, unable to consider the effect they may have had on our models. Finally, as with any case control study, significant associations cannot be assumed to be indicative of causal relationships. Randomized trial designs specifically targeting depression are needed to provide more direct evidence of causal relationships, and may now be warranted.

In conclusion, we observed that breast cancer inpatients were less likely to receive a clinical diagnosis of depression or anxiety compared with non-breast cancer inpatients. Patients who did receive a diagnosis of depression were less likely to die compared with those undiagnosed. If confirmed by future studies, our study results provide further indication that depending upon the definition and methods of assessment, depression and anxiety may be less common in breast cancer patients than previously thought, and suggest the need for additional research to better understand effects on survival.
